# Mapping of the Quantitative Trait Loci and Candidate Genes Associated With Iron Efficiency in Maize

**DOI:** 10.3389/fpls.2022.855572

**Published:** 2022-04-22

**Authors:** Jianqin Xu, Xiaoxin Qin, Huaqing Zhu, Fanjun Chen, Xiuyi Fu, Futong Yu

**Affiliations:** ^1^Key Laboratory of Plant-Soil Interaction (MOE), Centre for Resources, Environment and Food Security, College of Resources and Environmental Sciences, China Agricultural University, Beijing, China; ^2^Key Laboratory of Maize DNA Fingerprinting and Molecular Breeding, Maize Research Center, Beijing Academy of Agriculture and Forestry Science, Beijing, China

**Keywords:** maize (*Zea mays* L.), iron (Fe) deficiency tolerance, quantitative trait loci (QTL), iron (Fe) acquisition strategies, candidate genes

## Abstract

Iron (Fe) is a mineral micronutrient for plants, and Fe deficiency is a major abiotic stress in crop production because of its low solubility under aerobic and alkaline conditions. In this study, 18 maize inbred lines were used to preliminarily illustrate the physiological mechanism underlying Fe deficiency tolerance. Then biparental linkage analysis was performed to identify the quantitative trait loci (QTLs) and candidate genes associated with Fe deficiency tolerance using the recombinant inbred line (RIL) population derived from the most Fe-efficient (Ye478) and Fe-inefficient (Wu312) inbred lines. A total of 24 QTLs was identified under different Fe nutritional status in the Ye478 × Wu312 RIL population, explaining 6.1–26.6% of phenotypic variation, and ten candidate genes were identified. Plants have evolved two distinct mechanisms to solubilize and transport Fe to acclimate to Fe deficiency, including reduction-based strategy (strategy I) and chelation-based strategy (strategy II), and maize uses strategy II. However, not only genes involved in Fe homeostasis verified in strategy II plants (strategy II genes), which included *ZmYS1*, *ZmYS3*, and *ZmTOM2*, but also several genes associated with Fe homeostasis in strategy I plants (strategy I genes) were identified, including *ZmFIT*, *ZmPYE*, *ZmILR3*, *ZmBTS*, and *ZmEIN2*. Furthermore, strategy II gene *ZmYS1* and strategy I gene *ZmBTS* were significantly upregulated in the Fe-deficient roots and shoots of maize inbred lines, and responded to Fe deficiency more in shoots than in roots. Under Fe deficiency, greater upregulations of *ZmYS1* and *ZmBTS* were observed in Fe-efficient parent Ye478, not in Fe-inefficient parent Wu312. Beyond that, *ZmEIN2* and *ZmILR3*, were found to be Fe deficiency-inducible in the shoots. These findings indicate that these candidate genes may be associated with Fe deficiency tolerance in maize. This study demonstrates the use of natural variation to identify important Fe deficiency-regulated genes and provides further insights for understanding the response to Fe deficiency stress in maize.

## Introduction

Iron (Fe) plays an essential role in plant growth and development. It functions in various important processes, including photosynthesis, respiration, and chlorophyll biosynthesis, and it is a component in heme, Fe-sulfur cluster, and other Fe-binding sites (Kobayashi and Nishizawa, 2012). Meanwhile, it is a limiting factor for plant growth on approximately 30% of the world’s arable lands (Hindt and Guerinot, 2012). Fe deficiency is prevalent on calcareous soil and leads to a substantial decrease in crop yield (Briat et al., 2015). For example, peanuts grown on calcareous soil lead to yield losses of 20%, and losses can exceed 25% for several other crops, including cereals, legumes, vegetables, and fruit trees (Prasad, 2003). Fe deficiency can lead to reduced crop yield and quality, which in turn affects the immune and nervous systems and the mental development of humans who consume these crops (Li S. et al., 2019). Maize is one of the world’s important food crop, providing at least 30% of food calories to more than 4.5 billion people in 94 developing countries (Shiferaw et al., 2011). Therefore, breeding and promotion of Fe-efficient maize cultivars represent a biofortification strategy to alleviate Fe deficiency anemia and have a major impact on food security (Glahn et al., 2019).

Plants have evolved two distinct strategies to solubilize and transport Fe to acclimate Fe deficiency stress conditions, namely, the reduction strategy of non-graminaceous plants (strategy I) and the chelation strategy of graminaceous plants (strategy II) (Kobayashi and Nishizawa, 2012). Among them, in order to adapt to the fluctuation of Fe oxidation status and solubility in the field environment, rice, which is also a strategy II plant like maize, can use either strategy I or II to uptake Fe(II) or Fe(III) (Wang et al., 2020). The iron-related bHLH transcription factor 2 (IRO2), which is identified as a key regulator in rice, regulates the expression of phytosiderophore biosynthesis genes, including *OsNAS1*, *OsNAS2*, *OsNAAT1*, *OsDMAS1*, and *OsYSL15* encoding the Fe(III)-DMA transporter (Cheng et al., 2007; Ogo et al., 2007; Li Q. et al., 2019). OsFIT (also known as OsbHLH156) and OsIRO2 form a functional transcription activation complex to initiate the expression of Fe uptake genes (Liang et al., 2020). *In planta*, uptake of Fe(II) for strategy II plants has so far only been confirmed in rice using tracer experiments (Selby-Pham et al., 2017). The epidermis and exodermis of rice roots express various Fe(II) transporters in the plasma membrane, including OsIRT1, OsIRT2, OsNRAMP1, and OsNRAMP5 (Kobayashi et al., 2014; Ogo et al., 2014). There are two *FRO2-like* genes (*OsFRO1*, *OsFRO2*) and the phenolic efflux transporter (OsPEZ2), in the rice genome (Li Q. et al., 2019). However, their effect may be limited under Fe deficiency. This is likely because paddy rice has adapted to anaerobic conditions in which Fe(II) is abundant, making direct uptake without active ferric-chelate reduction sufficient for Fe acquisition (Kobayashi et al., 2014).

OsIDEF1 and 2 (IDE binding factor), belonging to the ABI3/VP1 and NAC plant-specific transcription factor families, respectively, have also been identified as positive regulators of the Fe deficiency response (Kobayashi et al., 2007). Under low Fe stress condition, OsIBP1.1 and OsIBP1.2 are Bowman-Birk trypsin inhibitors, which can interact with IDEF1 and thus prevent the 26S proteasome-mediated degradation of IDEF1 (Li Q. et al., 2019). *OsIDEF1* regulates the majority of known Fe uptake/utilization-related genes under the early stages of Fe deficiency, such as *OsYSL15*, *OsYSL2*, *OsIRT1*, *OsIRO2*, *OsNAS1*, *OsNAS2*, and *OsNAS3* (Kobayashi et al., 2009). In contrast, IDEF2 might not shift its target genes during different stages of Fe deficiency stress (Kobayashi et al., 2010). Moreover, it has been demonstrated that ethylene is involved in the Fe deficiency response in rice, but not in barley (Wu et al., 2011). In addition to rice, *SAMS* and *MTK* genes are also upregulated under Fe deficiency in some strategy II species, such as maize (Li et al., 2014). Rice is often grown in flooded paddies where Fe has the tendency to be reduced to the more bioavailable Fe(II) form (Selby-Pham et al., 2017). Therefore, this study also used a continuous and stable supply of Fe(II) to study whether important genes that regulate Fe homeostasis in strategy I plants could be identified in maize.

According to the research results of strategy I plants, gene regulatory network on Fe homeostasis is an important evolutionary product in plants for coping with fluctuating environments. FIT (FRU/AtbHLH29/FIT1), the FER ortholog in *Arabidopsis*, is able to complement the Fe deficiency response defects of mutant *T3238fer* (Wang et al., 2013). To exert the function of FIT, one or more subgroup Ib bHLH proteins (Heim et al., 2003), bHLH038, bHLH039, bHLH100, and bHLH101, needs to be present and form heterodimers for the induction to take place (Yuan et al., 2008). In addition, studies have demonstrated that the PYE regulatory network has a role in assuring the redistribution of already imported Fe (Long et al., 2010). PYE-targeting key genes implicated in metal homeostasis (including NAS4, FRO6, and ZIF1) play important roles in root growth under Fe deficiency (Kobayashi and Nishizawa, 2012).

Studies have shown that plant hormones and a variety of signal molecules are also involved in the response of plants to Fe deficiency stress. In maize mutants defective in Fe uptake, NO can revert chlorosis (Graziano and Lamattina, 2005). Under Fe limitation, a rapid and sustained NO accumulation is shown to be triggered in tomato plants, and the scavenging of NO leads to defects in the induction of *FER*/*FIT* and their downstream *IRT* and *FRO* genes (Chen et al., 2010). *GROWTH REGULATING FACTOR 11* (*GRF11*), which encodes a 14-3-3 protein, has been shown to act downstream of NO in promoting FIT transcription (Ivanov et al., 2012). Several studies have indicated that ethylene production increases under Fe deficiency in the roots of several strategy I plants, and ethylene is able to upregulate Fe acquisition (García et al., 2010; Lingam et al., 2011). Plant treatment with ethylene inhibitors abolishes some of the Fe deficiency responses and diminished the mRNA levels of *FRO2* and *IRT1* (Lucena et al., 2006). Ethylene and NO both require Fe-deficient condition to induce Fe-acquisition genes, and each one influences the production of the other (García et al., 2011). Unlike the signal molecules ethylene and NO, cytokinin, brassinosteroids, and jasmonate negatively regulate *Arabidopsis IRT1*, *FRO2*, and *FIT* expression through distinct pathways (Séguéla et al., 2008).

To date, for maize, most studies using genome-wide association studies or quantitative trait locus mapping approaches, have focused on Fe concentration in seeds and leaves (Lung’aho et al., 2011; Zdunić et al., 2014; Gu et al., 2015; Hindu et al., 2018; Ma et al., 2021), and only two reports concentrate on Fe homeostasis (Benke et al., 2014, 2015). In this study, we aimed to (1) screen out the most Fe-efficient and Fe-inefficient inbred lines to Fe deficiency stress among 18 inbred lines and preliminarily illustrate physiological mechanism underlying Fe deficiency tolerance; (2) identify important loci and candidate genes associated with the tolerance to Fe deficiency by biparental linkage analysis; and (3) analyze the expression pattern of the candidate genes involved in strategies I and II Fe acquisition.

## Materials and Methods

### Plant Material and Experimental Design

#### Experiment 1: Variations in Tolerance to Fe Deficiency of 18 Maize Inbred Lines

**FIGURE 1:**
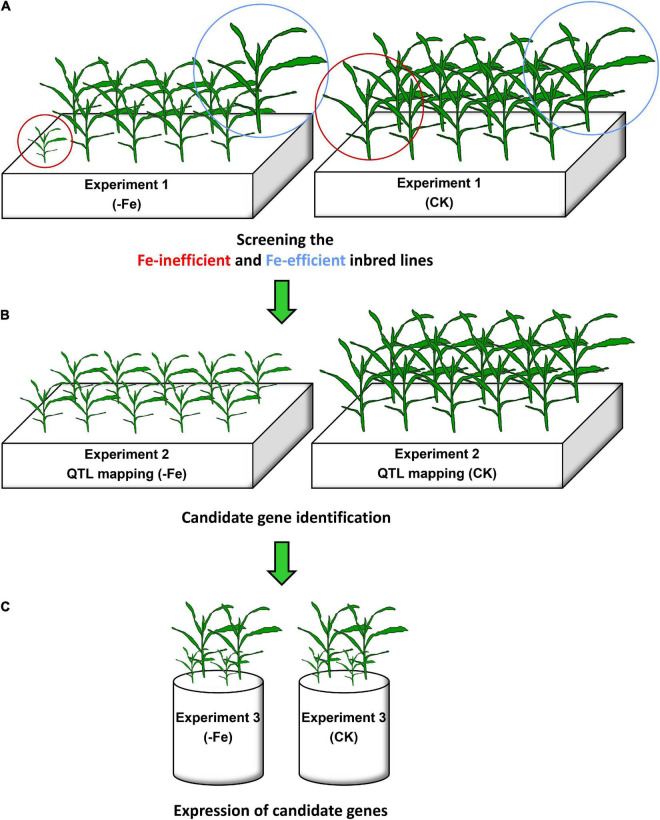
Experimental setup in this study. **(A)** In Experiment 1, the most Fe-inefficient and the most Fe-efficient inbred lines among the 18 maize inbred lines were screened out under Fe-deficient (–Fe) and Fe-sufficient (CK) conditions. **(B)** In Experiment 2, quantitative trait locus (QTL) analysis was performed in the –Fe and CK treatments, and candidate genes were further identified within these loci. **(C)** In Experiment 3, the expression of candidate genes in the shoots and roots of the most Fe-inefficient and the most Fe-efficient inbred lines was analyzed under Fe-deficient and Fe-sufficient conditions.

Eighteen maize inbred lines (i.e., Ye478, Chang7-2, KUI3, B73, K22, By815, X178, SC55, Zong3, Mo17, DE3, Dan340, Yu87-1, CI7, B77, Zheng58, HuangC, and Wu312) that have generated linkage populations among themselves were grown hydroponically in a 40 L container under Fe-deficient [−Fe1: 0.6 μmol L^–1^ Fe(II)-2,2′-bipyridyl] and Fe-sufficient [CK: 350 μmol L^–1^ Fe(II)-ethylene diamine tetraacetic acid (EDTA)] conditions (Figure 1A). Each treatment contained three replications.

#### Experiment 2: Quantitative Trait Loci Analysis for Fe Deficiency Tolerance in Maize

For quantitative trait loci (QTL) analysis, a recombinant inbred line (RIL) population consisting of 218 lines were derived from Fe-efficient inbred line Ye478 (female parent) and Fe-inefficient inbred line Wu312 (male parent) selected from Experiment 1. The genetic linkage map has a total length of 2,084 cm consisting of 184 polymorphic markers, as described by Liu et al. (2011). Two Fe-deficient and one Fe-sufficient treatments [−Fe1: 0.6 μmol L^–1^ Fe(II)-2,2′-bipyridyl, −Fe2: 0.06 μmol L^–1^ Fe(II)-2,2′-bipyridyl, CK: 350 μmol L^–1^ Fe(II)-EDTA] were set up (Figure 1B). Each treatment contained three replications.

#### Experiment 3: Expression of Candidate Genes

Fe-inefficient parent Wu312 and Fe-efficient parent Ye478 of Experiment 2 were hydroponically grown under Fe-deficient (−Fe) and Fe-sufficient (CK) conditions for 14 days (Figure 1C). Candidate genes selected from Experiment 2 were expressed in the shoots and roots of Wu312 and Ye478, including *ZmYS1* (GRMZM2G156599), *ZmBTS* (GRMZM2G320399), *ZmEIN2* (GRMZM2G068217), and *ZmILR3* (GRMZM2G093744). Each treatment contained three biological replications. Three technical replications were performed for each biological replication.

### Plant Growth

Maize seeds were sterilized for 30 min in a 10% solution of H_2_O_2_, washed with distilled water and soaked in saturated CaSO_4_ for 10 h, and then germinated on moist filter paper in the dark at room temperature. Two days later, the germinated seeds were wrapped in moist filter paper roll and grown. At the stage of two visible leaves, the seedlings were selected and transferred into a 40 L black container. The pH of the solution was set at 5.5–6.0. The adjusted Hoagland nutrient solution contained (mmol L^–1^) 0.5 NH_4_NO_3_, 0.5 CaCl_2_, 1.5 Ca(NO_3_)_2_, 0.75 K_2_SO_4_, 0.65 MgSO_4_, 0.1 KCl, 0.25 KH_2_PO_4_, 1.0 × 10^–3^ H_3_BO_3_, 0.01 Zn-EDTA, 8.0 × 10^–3^ CuSO_4_, 1.2 × 10^–2^ MnSO_4_, 4.0 × 10^–5^ (NH_4_)Mo_7_O_24_, 4.0 × 10^–3^ NiCl.

When Fe(II) (FeSO_4_.7H_2_O) was only added to the Hoagland nutrient solution, the rapid precipitation of non-chelated Fe(II) has been observed within a few hours of treatment (Regon et al., 2021). The competing cations, such as Ca(II) and Mg(II), may displace Fe(III) from some chelates (Cantera et al., 2002). Compared with EDTA, [*N,N′*-ethylenedi-amine-di-(*o*-hydroxyphenylacetic acid) (EDDHA)], and Diethylenetriaminepentaacetic acid (DTPA), 2,2′-bipyridyl, which is a lipophilic Fe(II)-specific chelator, can maintain a continuous supply of Fe(II) (Iwatsuki et al., 2008). In addition, previous studies have found that low Fe stress can cause root decay and leaf chlorosis and senescence in maize. 2,2′-Bipyridyl can inhibit leaf senescence by chelation of Fe(II) ions involved in the synthesis of a protein required for senescence and can also inhibit the reproduction of bacteria in the water (Knee, 1996). Therefore, this study used 2,2′-bipyridyl to continuously supply Fe(II) and investigated the response of maize to low Fe stress.

All experiments were conducted in the growth chamber under strictly controlled environments. The growth chamber condition was set as a 14-h light period from 8:00 a.m. to 10:00 p.m. at 28°C and a 10-h dark period at 22°C. The average light intensity measured at canopy was 350 μmol m^–2^ s^–1^.

### Data Collection

Experiments 1 and 2 were terminated 21 days after transplanting. During the period from 9:00 to 11:00 a.m., soil plant analysis development (SPAD) values of the youngest fully expanded leaf were measured on the 1/3 part from the leaf base three times using SPAD-502 leaf chlorophyll meter. The mean of three observed values was recorded for each plant. The plant height and root length were recorded, were heat-treated at 105°C for 30 min and dried at 75°C until constant weight. Dried shoots for each plant sample were separately ground into a fine powder, and 0.3000 g powder was digested with HNO_3_-H_2_O_2_ in a microwave-accelerated reaction system (CEM, Matthews, NC, United States). The concentrations of Fe, Cu, and Zn in the digested solutions were determined by inductively coupled plasma atomic emission spectroscopy (OPTIMA 3300 DV, PerkinElmer, United States). Fe efficiency, relative ratios of root to shoot (R/S) efficiency, nutrient contents, and uptake efficiency were estimated using the following equations from (1) to (4), respectively.

(1) Fe efficiency = dry weight (−Fe)/dry weight (CK)
(2) Relative ratios of R/S = R/S (−Fe)/R/S (CK)
(3) Nutrient content = concentration × dry weight
(4) Uptake efficiency = (total nutrient content)/(root dry weight)

### Statistical Analysis

The means for each trait were compared using one-way ANOVA at a 0.05 level of probability followed by the least significant difference test of SPSS 20.0. The linear mixed effect function lmer in the lme4 package of R version 3.1.1 was fitted to each RIL to obtain the best linear unbiased prediction (BLUP) value for each trait. These variance components were considered to calculate the broad-sense heritability as *H*^2^ = σ*_*G*_*^2^/(σ*_*G*_*^2^ + σ*_*GE*_*^2^*/e* + σ*_*E*_*^2^*/re*), where σ*_*G*_*^2^ is genetic variance, σ*_*GE*_*^2^ is the interaction of genotype and environment, σ*_*E*_*^2^ is the residual error, while *e* and *r* are the number of environments and replications, respectively. The ratio of −Fe to CK (−Fe/CK) for each trait was calculated by the BLUP of each line in the RIL population.

### Quantitative Trait Loci Analysis

The QTL identification was performed using composite interval mapping in Windows QTL Cartographer version 2.5. Model 6 was selected for detecting QTLs and estimating their effects. The threshold logarithm of odds (LOD) values to declare the putative QTLs were estimated by permutation tests with a minimum of 1,000 replicates at a significant level of *p* < 0.05 (LOD = 3.0). The confidence interval for each QTL was determined using the 1-LOD interval method.

### Annotation of Candidate Genes

The functional descriptions were identified using the maize B73 reference genome assembly version 2 available on the MaizeGDB Database^1^ and Gramene Database.^2^

### RNA Extraction and Gene Expression Quantification

In experiment 3, total RNA was extracted from the shoots and roots of plants using the Total RNA Extraction Kit (TIANGEN, China). The cDNA was synthesized in accordance with Fast Quant RT Super Mix Reverse Transcription Kit instructions (TransGene, Beijing, China). Quantitative real-time PCR was performed using SYBR Green Real-time RT-PCR (Takara) and an ABI7500 Fast Real-Time PCR System (Applied Biosystems). The primers used for real-time PCR are shown in Supplementary Table 1. The relative gene expression level was calculated using the 2^–ΔΔCt^ method. Each real-time PCR experiment contained three technical replicates.

## Results

### Variations in Fe Deficiency Tolerance of 18 Maize Inbred Lines

**FIGURE 2:**
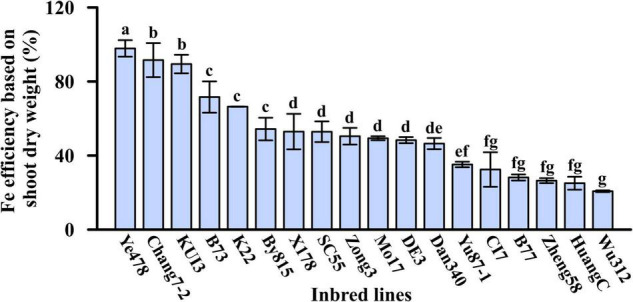
Fe efficiency based on shoot dry weight (%) of the 18 maize inbred lines in Experiment 1. Different letters indicate a significant difference among the 18 inbred lines at *p* < 0.05.

The relative shoot growth is the most suitable parameter for evaluating the ability of Fe deficiency tolerance in crops (MacNair, 1993; Rengel and Römheld, 2000). In this study, Fe efficiency refers to the ratio of shoot dry weight under Fe-deficient condition to the shoot dry weight under Fe-sufficient condition. Eighteen maize inbred lines responded differentially to Fe deficiency, and their Fe efficiencies varied from 21 to 98%. Among them, Ye478 and Chang7-2, Wu312 and HuangC obtained the highest and lowest Fe efficiencies, respectively (Figure 2).

**FIGURE 3:**
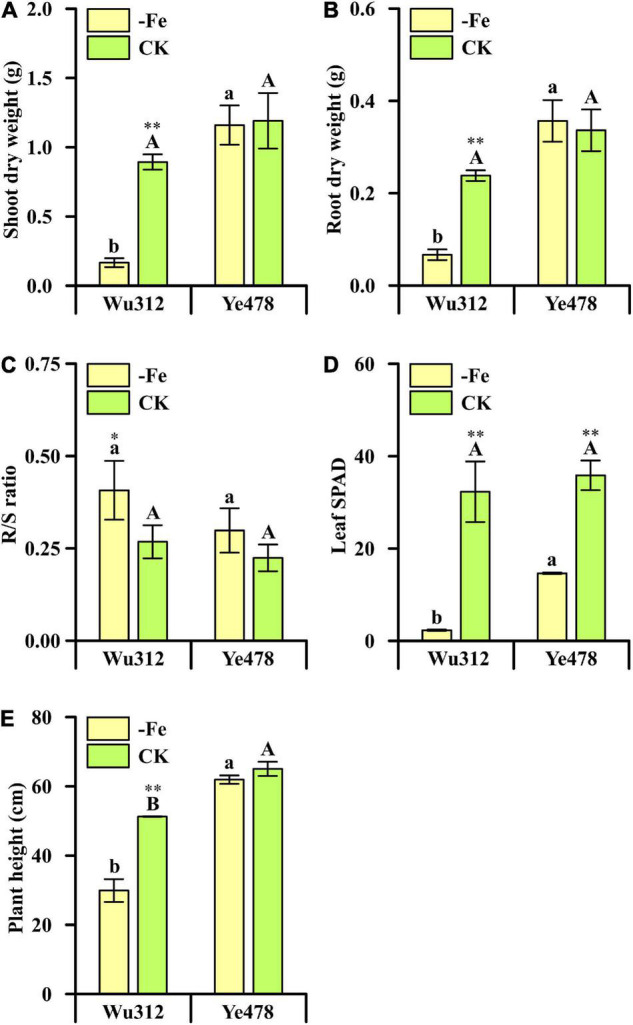
Phenotypic difference of each trait between the most Fe-sensitive (Wu312) and Fe-tolerant inbred lines (Ye478) under Fe-deficient [–Fe: 0.6 μmol L^–1^ Fe(II)-2,2′-bipyridyl] and Fe-sufficient [CK: 350 μmol L–^1^ Fe(II)-EDTA] conditions in Experiment 1. **(A)** Shoot and **(B)** root dry weight; **(C)** R/S ratio; **(D)** leaf SPAD; **(E)** plant height. Different uppercase (lowercase) letters indicate significant differences between Ye478 and Wu312 in the –Fe (CK) treatments at *p* < 0.05. * and ^**^ indicate significant differences between the –Fe and CK treatments at *p* < 0.05 and *p* < 0.01, respectively.

**FIGURE 4:**
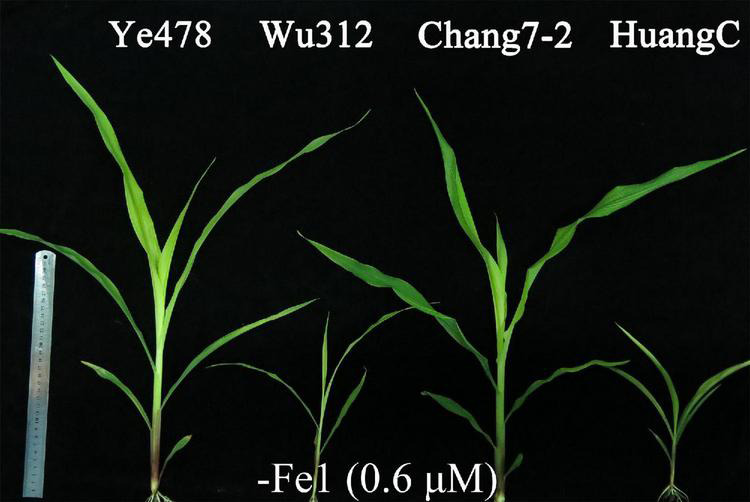
Shoots of maize inbred line Ye478, Wu312, Chang7-2, and HuangC (from left to right) under Fe-deficient condition [–Fe1: 0.6 μmol L^–1^ Fe(II)-2,2′-bipyridyl] in Experiment 1.

Phenotypic variations of the two most contrasting inbred lines (Wu312 and Ye478) are shown in Figure 3. Fe deficiency decreased the shoot dry weights of Wu312 and HuangC by 79 and 75%, respectively; and reduced their root weights by more than 75% (Figures 3A,B and Supplementary Table 2). However, low Fe supply had no significant effects on the shoot and root dry weights, R/S ratio, and plant height of Ye478 (Figures 3A–C,E and Supplementary Tables 2, 3). In addition, more decreases were observed in the leaf SPAD of Wu312 than that of Ye478 under deficient Fe supply (Figure 3D). Under Fe-deficient condition, Ye478 and Chang7-2 developed six leaves with one sprout, and the plant growth and development of Wu312 and HuangC was strongly inhibited (Figure 4).

Fe deficiency stress leads to an increase in R/S ratio, indicating a reduction in shoot and, to a lesser extent, root growth in plants (Cianzio and Voss, 1994). Increases in R/S ratios induced by Fe deficiency were more in Wu312 and HuangC than in Ye478 and Chang7-2 (Figure 3C and Supplementary Table 2), indicating a relatively small decrease in root growth compared with shoot growth. These results implicated a fine regulation of photo-assimilate distribution between organs, reflecting a preferential investment of biomass from shoots to roots at low nutrient availability (Yousfi et al., 2009). Although Fe deficiency stress leads to a reduction in root growth, morphological alteration of the root may contribute to Fe-deficiency-induced physiological responses, such as the development of lateral roots (Li et al., 2014).

Under Fe-deficient condition, shoot and root dry weights of Ye478 were more than five times higher than those of Wu312 (Figures 3A,B and Supplementary Table 2). Besides, leaf SPAD and plant height of Ye478 were higher than those of Wu312 (Figures 3D,E and Supplementary Table 3). These indicate that Ye478 had a higher chlorophyll content in leaves, higher plant height, and biomass accumulation in response to Fe-deficiency stress compared with Wu312. Taken together, Ye478 and Wu312 were screened out to be the most Fe-efficient and Fe-inefficient maize inbred lines, respectively, allowing for further genetic molecular work.

### Variations of Fe, Cu, and Zn in Maize Under Fe Deficiency

**FIGURE 5:**
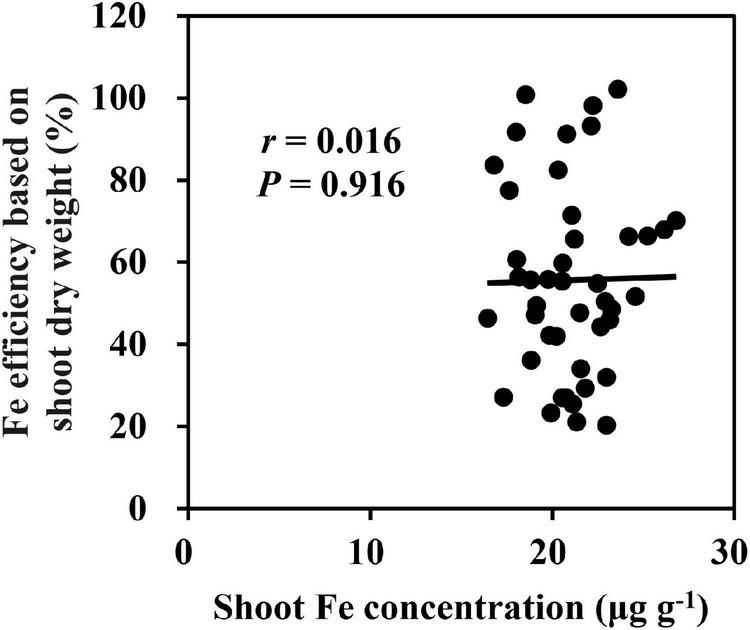
Relationship between Fe efficiency based on shoot dry weight and shoot Fe concentration under Fe deficiency.

Under low Fe supply, the average of shoot Fe concentration for 18 inbred lines was significantly decreased from 81.7 to 21.0 μg g^–1^, which was significantly lower than that under Fe-sufficient condition (Supplementary Figure 1). Besides, we found that shoot Fe concentration of 18 inbred lines was not correlated to Fe efficiency (*p* = 0.916) (Figure 5), indicating that the shoot Fe concentration was not a sensitive parameter to characterize Fe deficiency tolerance. There was no significant difference in shoot and root dry weights of Ye478 between the −Fe and CK treatments, but a great reduction was observed in shoot Fe concentration, implying that Fe-efficient inbred line Ye478 made the most of Fe for plant growth under Fe-deficient condition. A slight increase in shoot Cu concentration and a dramatic increase in shoot Zn concentration were observed under Fe-deficient condition (Supplementary Figure 2). Furthermore, Cu and Zn uptake efficiency was increased by more than sixfold by Fe deficiency (Supplementary Figure 3).

### Quantitative Trait Loci Analysis of Fe Deficiency Tolerance in Maize

#### Phenotypic Data Analysis

**FIGURE 6:**
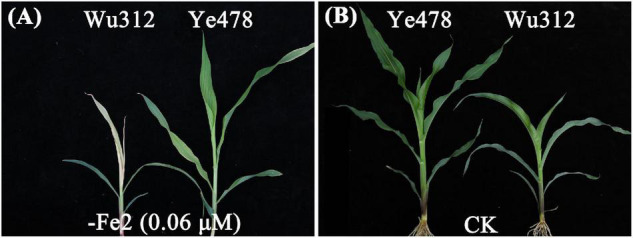
Shoots of Fe-inefficient parent Wu312 and Fe-efficient parent Ye478 under Fe-deficient and Fe-sufficient conditions in Experiment 2. **(A)** –Fe2, 0.06 μmol L^–1^ Fe(II)-2,2′-bipyridyl; **(B)** CK, 350 μmol L^–1^ Fe(II)-EDTA.

The phenotypic means of each trait for the parents and their RIL population are shown in Supplementary Table 4. For QTL analysis, two supply levels of Fe-deficiency stress [−Fe1: 0.6 μmol L^–1^ Fe(II)-2,2′-bipyridyl; −Fe2: 0.06 μmol L^–1^ Fe(II)-2,2′-bipyridyl] were designed to identify the loci associated with Fe deficiency tolerance. Compared with −Fe1 deficiency stress, −Fe2 stress caused a more severe Fe deficiency on both Wu312 and Ye478. Under −Fe2 stress, Fe-inefficient parent Wu312 showed severe Fe-deficient chlorosis in young leaves and tended to be dead, and Ye478 tended to develop the fifth leaf and displayed a few chloroses in young leaves compared with Fe-sufficient plants (Figure 6).

**FIGURE 7:**
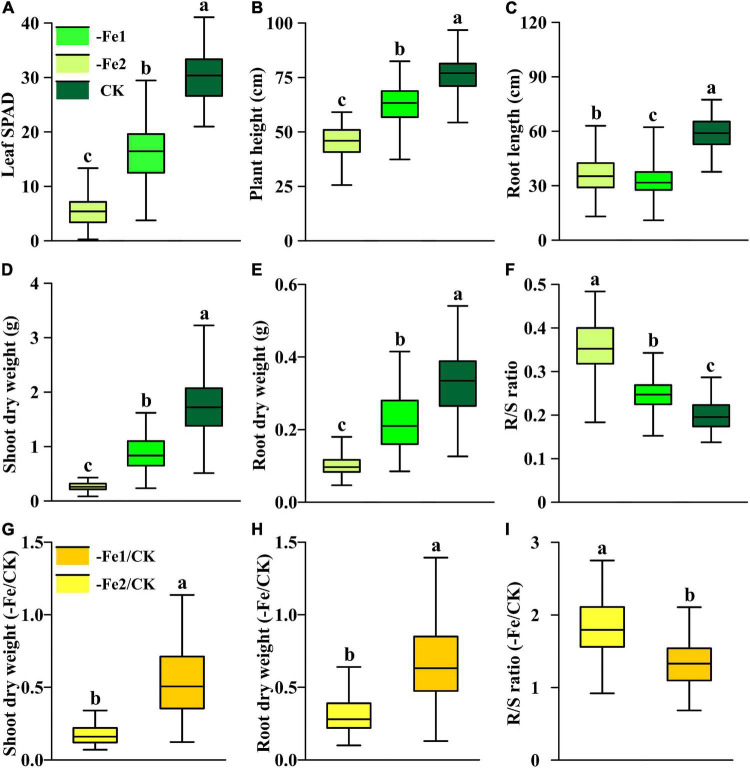
Phenotypic distribution of each trait under different Fe supply levels (–Fe1, –Fe2, CK, –Fe1/CK, –Fe2/CK) in the Ye478 × Wu312 RIL population. **(A)** Leaf SPAD; **(B)** plant height; **(C)** root length; **(D,G)** shoot dry weight; **(E,H)** root dry weight; **(F,I)** R/S ratio. The solid line in each box represents the median values. The top and bottom edges of each box represent the 75th and 25th percentiles, respectively. Different letters indicate a significant difference between different treatments at *p* < 0.05.

Both −Fe1 and −Fe2 deficiency stresses resulted in damage in plant growth, including leaf chlorophyll content, plant height, shoot and root biomass accumulation, but led to increases in R/S ratios of Fe-deficient plants (Figure 7). Under −Fe1 and −Fe2 deficiency, Ye478 exhibited advantages in leaf SPAD, plant height, shoot and root dry weights but not in root length. There were greater differences of leaf SPAD, shoot and root dry weights between Ye478 and Wu312 under −Fe1 supply in comparison with −Fe2 supply (Supplementary Table 4). Under −Fe1 supply, Fe efficiency for Wu312 was no more than 30%; nevertheless, Ye478 obtained the highest Fe efficiency (>97%). In the −Fe2 treatment, Fe efficiencies of Wu312 and Ye478 were very low, ranging from 13 to 33%. On the contrary, root length for two parents under −Fe2 stress was longer than that in the −Fe1 treatment, suggesting that plants may develop longer root length to facilitate more nutrient uptake in response to more severe Fe deficiency. The signaling pathways triggering morphological changes in root induced by Fe deficiency may be related to chemical signaling, especially auxin, which is strongly involved in root development (Nacry et al., 2005; Jin et al., 2007).

All the traits of the RIL population varied widely in different treatments (Supplementary Table 4). The coefficients of variance for each trait changed from 11.5 to 50.0% in the Ye478 × Wu312 RIL population. A more severe Fe deficiency of −Fe2 led to a greater reduction in leaf SPAD, plant height, shoot and root dry weights of the population, while resulting in a significant enhancement in root length and R/S ratio, compared with the Fe-deficient supply of −Fe1 (Figures 7A–E). To identify the loci associated with Fe efficiency, we used all traits in the −Fe treatments (−Fe1, −Fe2), shoot and root dry weights, and R/S ratio in the −Fe/CK treatments (−Fe1/CK, −Fe2/CK) to perform QTL analysis. Under the −Fe2/CK condition, the shoot and root dry weights varied below 0.5 and 0.8, respectively, which were substantially lower than those in the −Fe1/CK treatment (Figures 7G,H). Furthermore, a greater increase in R/S ratio induced by low Fe stress was observed under −Fe2 deficiency, not under −Fe1 deficiency (Figure 7I). Furthermore, broad-sense heritabilities for all the traits in the −Fe or the −Fe/CK treatments in the population were higher than 64% (Supplementary Table 4), indicating that these traits associated with Fe deficiency tolerance were genetically controlled.

#### Quantitative Trait Loci Detection

**FIGURE 8:**
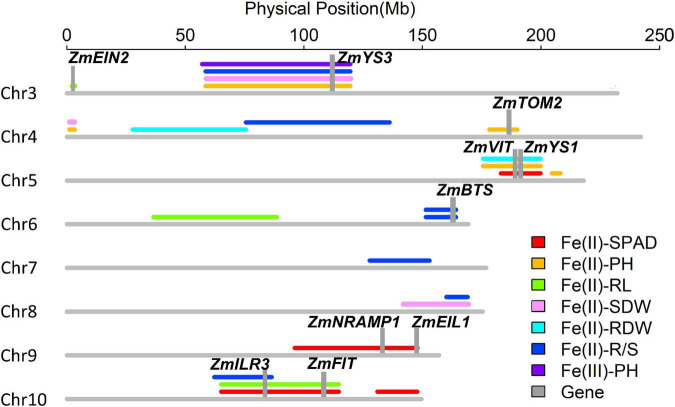
Twenty-four quantitative trait loci controlling leaf SPAD (SPAD), plant height (PH), root length (RL), shoot (SDW), root (RDW) dry weight, and R/S ratio (R/S) were detected on chromosomes 3, 4, 5, 6, 7, 8, 9, and 10 in the Ye478 × Wu312 RIL population. Ten candidate genes (gray columns) identified within these loci were considered to be associated with Fe deficiency tolerance, containing *ZmEIN2* (GRMZM2G068217), *ZmYS3* (GRMZM2G063306), *ZmTOM2* (GRMZM5G877788), *ZmVIT* (GRMZM2G409358), *ZmYS1* (GRMZM2G156599), *ZmBTS* (GRMZM2G320399), *ZmNRAMP1* (GRMZM2G178190), *ZmEIL1* (GRMZM2G317584), *ZmILR3* (GRMZM2G093744), and *ZmFIT* (GRMZM2G313756).

A total of 24 QTLs controlling six traits was identified at an empirical threshold LOD value of 3.0 estimated by 1,000 permutation tests, explaining 6.1–26.6% of phenotypic variation (Figure 8 and Supplementary Table 5). Four loci [*qFe(II)-SPAD5-1, qFe(II)-SPAD10-1, qFe(II)-SPAD10-2* and *qFe(II)-SPAD9-1*] for leaf SPAD were identified on chromosomes 5, 9, and 10 under −Fe2 deficiency stress, accounting for 6.1–25.6% of phenotypic variation. The third largest effect QTL *qFe(II)-SPAD10-1* controlling leaf SPAD was detected on chromosome 10 under −Fe1 deficiency stress, accounting for 25.1% of phenotypic variation. Alleles from parent Wu312 increased leaf SPAD by 3.1 at this locus.

Five QTLs [*qFe(II)-PH3-1*, *qFe(II)-PH4-1*, *qFe(II)-PH4-2*, *qFe(II)-PH5-1*, and *qFe(II)-PH5-2*] for plant height were identified on chromosomes 3, 4, and 5, explaining 15.2–24.2% of phenotypic variation. Two QTLs on chromosome 4 were detected under Fe1 and Fe2 deficiency, respectively. The alleles from parent Wu312 contributed to increased plant height at these two loci. Two loci were mapped on chromosome 5, and one locus was identified on chromosome 3, accounting for 17.1–24.2% of phenotypic variation. Alleles from Yee478 increased plant height by 5.0 and 3.9 cm at *qFe(II)-PH5-1* and *qFe(II)-PH3-1*, respectively. Three loci [*qFe(II)-RL3-1*, *qFe(II)-RL6-1*, *qFe(II)-RL10-1*] for root length were identified on chromosomes 3, 6, and 10 and accounted for 18.6–26.6% of phenotypic variation. The largest effect QTL *qFe(II)-RL10-1* was detected under −Fe1 deficiency, and the allele from parent Wu312 enhanced the root length by 4.2 cm at this locus.

Three minor effect QTLs controlling shoot dry weight, two minor effect QTLs controlling root dry weight, and seven QTLs controlling R/S ratio were detected on chromosomes 3, 4, 5, 6, 7, 8, and 10, explaining 7.2–26.1% of phenotypic variation. The alleles from Ye478 contributed to enhanced root dry weight by 0.04 g at *qFe(II)-RDW4-1* and *qFe(II)-RDW5-1*. Seven QTLs controlling R/S ratio were identified, and the alleles from parent Ye478 contributed to increased R/S ratio. The second-largest effect QTL *qFe(II)-R/S4-1* contained the alleles from Ye478 enhancing the relative R/S ratio by 0.17.

### Quantitative Trait Loci Co-localization and Identification of Candidate Genes

A total of six QTL co-localizations were identified on chromosomes 3, 4, 5, 6, 8, and 10 in the Ye478 × Wu312 RIL population (Supplementary Table 6). On chromosome 3, the co-localization detected by three QTLs [*qFe(II)-PH3-1*, *qFe(II)-SDW3-2*, and *qFe(II)-R/S3-1*] was located at 58,480,342–119,639,144 bp. On chromosome 4, the region co-localized by *qFe(II)-PH4-2*, and *qFe(II)-SDW4-1* was located between 1,075,603 and 3,262,840 bp. The QTL co-localization detected by a large effect QTL *qFe(II)-PH5-1* and two minor effect QTLs [*qFe(II)-SPAD5-1* and *qFe(II)-RDW5-1*] was located at 175,608,742–199,787,515 bp on chromosome 5. On chromosome 6 and 10, two co-localizations detected by large-effect QTLs were identified at 151,770,259–164,150,176 bp and 65,096,550–114,698,107 bp, respectively. The overlapped genomic regions detected by two minor QTLs were located at 160,098,642–169,054,438 bp on chromosome 8. Candidate genes were further identified within these genomic regions.

A total of 1,543 genes were identified on these six co-localizations: 179 genes on chromosome 3, 46 genes on chromosome 4, 361 genes on chromosome 5, 336 genes on chromosome 6, 236 genes on chromosome 8, and 385 genes on chromosome 10. The detailed information for each gene is shown in Supplementary Table 6. According to the functional descriptions of total 1,543 genes in *Arabidopsis* on MaizeGDB Database (see text footnote 1) and Gramene Database (see text footnote 2), maize homologs of the genes involved in strategies I and II Fe acquisition, Fe uptake, and transport in *Arabidopsis* and rice were identified and annotated. Among them, six candidate genes were proposed to be associated with Fe deficiency tolerance in maize, including *ZmYS1*, *ZmYS3*, *ZmVIT*, *ZmBTS*, *ZmILR3*, and *ZmFIT* (Table 1). According to previous reports on the molecular mechanism underlying Fe acquisition strategies and Fe homeostasis in other plants, another four candidate genes were identified in other three QTLs, including *ZmEIN2*, *ZmTOM2*, *ZmNRAMP1*, and *ZmEIL1* (Table 1).

**TABLE 1:** Candidate genes associated with Fe deficiency tolerance identified by linkage mapping in maize.

Chr	Trait	QTL	Gene ID	Position (bp)	Annotation
3	RL	*qFe(II)-RL3-1*	GRMZM2G068217	2,874,829–2,881,374	ZmEIN2—Ethylene insensitive 2
3	PH, SDW, R/S	*qFe(II)-PH3-1*, *qFe(II)-SDW3-1*, *qFe(II)-R/S3-1*	GRMZM2G063306	112,042,104–112,047,482	ZmYS3—Yellow stripe 3
4	PH	*qFe(II)-PH4-1*	GRMZM5G877788	186,480,512–186,484,517	ZmTOM2—Transporter of mugineic acid 2
5	SPAD, PH,	*qFe(II)-SPAD5-1*, *qFe(II)-PH5-1*,	GRMZM2G409358	189,865,490–189,866,191	ZmVIT—Vacuolar iron transporter (VIT) family protein
	RDW	*qFe(II)-RDW5-1*	GRMZM2G156599	190,674,766–190,677,896	ZmYS1—Yellow stripe 1
6	R/S	*qFe(II)-R/S6-1, qFe(II)-R/S6-2*	GRMZM2G320399	162,682,754–162,690,139	ZmBTS—Encodes BRUTUS (BTS), a putative E3 ligase protein
9	SPAD	*qFe(II)-SPAD9-1*	GRMZM2G178190	147,509,844–147,513,844	ZmNRAMP1—NRAMP metal ion transporter 1
9	SPAD	*qFe(II)-SPAD9-1*	GRMZM2G317584	133,215,979–133,219,767	ZmEIL1—ETHYLENE-INSENSITIVE3-like 1
10	SPAD, RL	*qFe(II)-SPAD10-1*, *qFe(II)-RL10-1*	GRMZM2G093744	83,592,659–83,595,951	ZmILR3—bHLH-transcription factor 162
			GRMZM2G313756	108,358,694–108,359,794	ZmFIT—FER-like regulator of iron uptake

*ZmYS1* (GRMZM2G156599) and *ZmYS3* (GRMZM2G063306) encoding yellow stripe family proteins were detected in the genomic region overlapped by *qFe(II)-SPAD5-1*, *qFe(II)-PH5-1*, *qFe(II)-RDW5-1* on chromosome 5 and *qFe(II)-PH3-1*, *qFe(II)-SDW3-1*, *qFe(II)-R/S3-1* on chromosome 3, respectively. *Yellow Stripe 1* is the first gene encoding an Fe(III)-phytosiderophore transporter at root surface in maize (Curie et al., 2001). *ZmTOM2* (GRMZM5G877788) encoding transporters of mugineic acid family phytosiderophores was located within the single locus *qFe(II)-PH4-1*.

Apart from strategy II genes, several candidate genes involved in Fe homeostasis in strategy I plants were also identified in other genomic regions. *ZmFIT* (GRMZM2G313756), which encodes FER-like Fe deficiency-induced transcriptional factor and may be the key for Fe homeostasis in strategy I plants, was identified within the overlapped region co-localized by *qFe(II)-SPAD10-1* and the largest effect QTL *qFe(II)-RL10-1* on chromosome 10. Beyond that, *ZmILR3* (GRMZM2G093744) encoding bHLH-transcription factor 162, was located in the QTL co-localization of two large-effect QTLs and a minor QTL on chromosome 10. *ZmEIN2* (GRMZM2G068217) encoding ETHYLENE-INSENSITIVE2 and *ZmEIL1* (GRMZM2G317584) encoding ETHYLENE-INSENSITIVE3-like transcription factor were identified, homologs of which play important roles in responses to ethylene and interact with FIT in strategy I plants. Furthermore, *ZmILR3*, together with *ZmBTS* (GRMZM2G320399) which encodes a putative E3 ligase protein and was identified in *qFe(II)-R/S6-1*, may be both involved in the PYE network that could be another transcriptional regulatory network in addition to the FIT network. It should be noted that no QTLs detected on the supply of Fe(III) reported by Xu et al. (2022), were co-localized with the QTLs containing strategy I genes in the current study, which only utilized Fe(II). Moreover, only strategy II gene *ZmYS3* was identified in the co-localization of one of our previously reported locus and three QTLs detected in this study.

In addition, two genes probably responsible for Fe transport were identified in two loci. *ZmVIT* (GRMZM2G409358) encoding vacuolar iron transporter (VIT) family protein was also located in the co-localization of *qFe(II)-SPAD5-1, qFe(II)-PH5-1*, and *qFe(II)-RDW5-1*. It is reported that OsVIT1 and OsVIT2 are responsible for transporting Fe across the tonoplast into the vacuole in rice (Kobayashi et al., 2014). *ZmNRAMP1* (GRMZM2G178190) encoding NRAMP metal ion transporter 1 in maize was located within *qFe(II)-SPAD9-1*.

### Expression Patterns of Candidate Genes in Different Tissues

**FIGURE 9:**
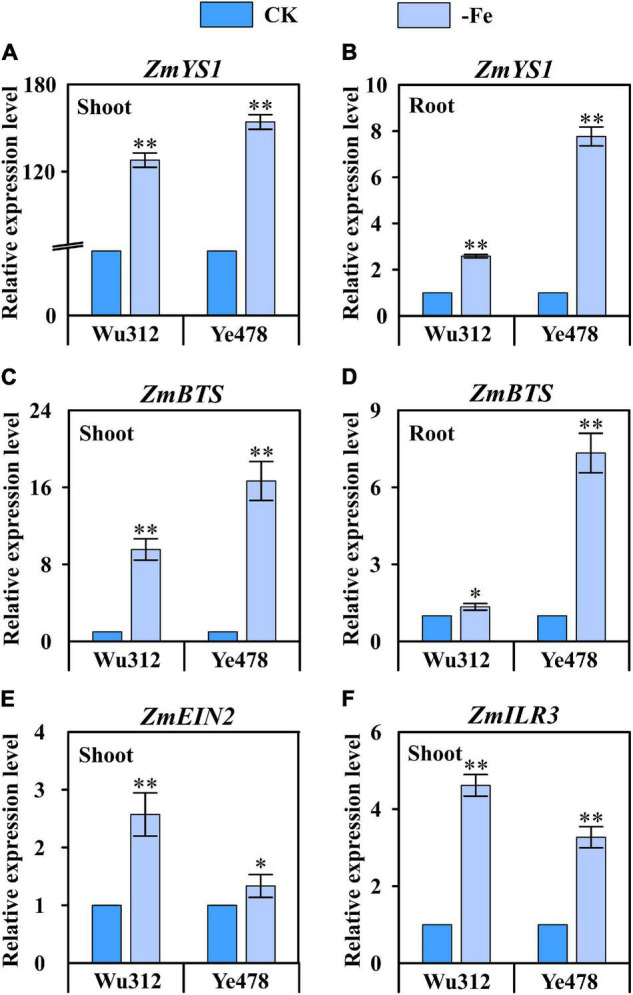
Relative expression level of four candidate genes in the shoots and roots of Fe-inefficient (Wu312) and Fe-efficient (Ye478) parents under Fe-deficient (–Fe) and Fe-sufficient (CK) conditions. **(A,B)** *ZmYS1* and **(C,D)** *ZmBTS* expression in the shoots and roots, **(E)** *ZmEIN2* and **(F)** *ZmILR3* expression in the shoots. * and ^**^ indicate significant difference between the –Fe and CK treatments at *p* < 0.05 and *p* < 0.01, respectively.

According to the QTL co-localization identified in this study, the relative expression levels of candidate genes *ZmYS1* (GRMZM2G156599), *ZmBTS* (GRMZM2G320399), *ZmEIN2* (GRMZM2G068217), and *ZmILR3* (GRMZM2G093744) were analyzed. *ZmYS1* was significantly upregulated in both roots and shoots under Fe-deficient condition (Figures 9A,B). Under Fe deficiency, *ZmYS1* was 128- and 154-fold upregulated in the shoots of Wu312 and Ye478, respectively (Figure 9A). *ZmYS1* was 2.6- and 7.8-fold upregulated in the roots of Wu312 and Ye478, respectively (Figure 9B). This indicates that the expression of *ZmYS1* responds to Fe deficiency stress more in shoots than in roots. Interestingly, the upregulation of *ZmBTS*, homologs of which are participating in Fe deficiency tolerance in strategy I plants, was more than twice higher in the shoots than in the roots (Figures 9C,D). It should be noted that the upregulation of *ZmYS1* and *ZmBTS* in both shoots and roots of Fe-efficient parent Ye478 was greater than that for Fe-inefficient parent Wu312, suggesting that *ZmYS1* and *ZmBTS* are not only involved in the mechanism on Fe deficiency tolerance, but also associated with the genotypic difference in Fe efficiency between Fe-efficient and Fe-inefficient maize inbred lines. *ZmEIN2* and *ZmILR3*, of which homologs are found to be important for strategy I Fe acquisition, were both markedly upregulated in the shoots of two parents in response to low Fe stress (Figures 9E,F). These findings further indicate that the genes involved in regulation of Fe homeostasis in strategy I plants may play important roles in tolerance to Fe deficiency stress in maize, which is considered a strategy II plant.

## Discussion

### Comparisons of Quantitative Trait Loci Identified in This Study With Previous Reports

To our limited knowledge, there are numerous linkage or association mapping studies concentrating on the concentrations of Fe and other metals in maize (Qin et al., 2012; Šimić et al., 2012; Jin et al., 2013; Zdunić et al., 2014; Gu et al., 2015; Zhang et al., 2017; Hindu et al., 2018; Ma et al., 2021), and only two reports focusing on the identification of loci associated with Fe homeostasis in maize (Benke et al., 2014, 2015). In addition, there are several reports on identifying the QTLs associated with plant height, shoot and root biomass accumulation, root traits under normal conditions, salt stress, and nutrient-deficient conditions rather than under Fe-deficient condition (Azevedo et al., 2015; Burton et al., 2015; Li et al., 2016; Wang et al., 2016; Luo et al., 2017). Therefore, the loci identified in these previous studies were compared with this study based on the physical position of each locus.

In total, 20 of 24 QTLs detected by linkage analysis have been found to be co-localized with the loci detected in previous researches (Supplementary Table 5). Among these loci, a total of 11 QTLs controlling six different traits in this study were co-localized with the loci controlling leaf SPAD, shoot and root dry weights, leaf necrosis, shoot water content under Fe-deficient and Fe-adequate conditions detected in the maize IBM population, and association population (Benke et al., 2014, 2015). Additionally, five QTLs associated with leaf SPAD, shoot dry weight, and R/S ratio detected in our study were co-localized with the QTLs associated with Fe concentration in grains, and copper (Cu) and magnesium (Mg) concentration in leaves detected by linkage or association analysis (Jin et al., 2013; Zdunić et al., 2014; Zhang et al., 2017; Hindu et al., 2018; Ma et al., 2021), suggesting that these co-localized QTL regions may have pleiotropic effects on Fe and other mineral concentrations of grains and leaves in maize.

In addition, a total of seven QTLs controlling leaf SPAD, plant height, root length, and root dry weight detected in this study were co-localized with the loci controlling plant height under salt stress or normal conditions reported by Luo et al. (2017). Besides, seven QTLs controlling six different traits identified in this work were overlapped with the loci controlling root morphology under low phosphorus (P) stress detected by Azevedo et al. (2015) and nitrogen (N) stress identified by Li et al. (2016). These findings indicate that these QTLs may harbor several genes with pleiotropic effect on plant height or root morphology under abiotic stress at maize seedling stage.

### Candidate Genes Maintain Fe Homeostasis Under Low-Fe Stress in Maize

In this study, *ZmYS1*, *ZmYS3*, and *ZmTOM2*, were identified within the genomic regions detected by QTL analysis. Strategy II plants acquire Fe through mugineic acid family phytosiderophores (MAs) (Bashir and Nishizawa, 2006). After being synthesized in root cells, MAs are secreted into the rhizosphere *via* transporter of mugineic acid family phytosiderophores (TOMs) (Li Q. et al., 2019). Finally, Fe(III)-MA complexes are taken up from the rhizosphere by transporter YS1 in maize (Curie et al., 2001; Inoue et al., 2009).

In this study, we confirmed that the expression of *ZmYS1* was remarkably induced by Fe deficiency in both shoots and roots (Figures 9A,B), which was in accordance with previous studies (Ueno et al., 2009; Nozoye et al., 2013; Wairich et al., 2019). Besides, our results showed that upregulation of *ZmYS1* induced by Fe deficiency was greater in the shoots than in the roots, which is consistent with the results reported by Nozoye et al. (2013). These outcomes are in line with the findings demonstrated by Ueno et al. (2009), who found that *ZmYS1* may be involved in both primary Fe acquisition and intracellular transport of Fe in maize. Besides, another homologous gene of *ZmYS1*, *OsYSL15* is found to encode an Fe-specific transporter that is not only responsible for Fe uptake from the rhizosphere but also for phloem transport of Fe *via* Fe(III)-DMA (Inoue et al., 2009; Lee et al., 2009). These findings may further support the possibility of dual roles for ZmYS1:Fe uptake from soil and Fe distribution within plants.

The expression levels of the genes involved in MAs biosynthesis (*ZmDMAS1*), Fe acquisition, uptake, and transport (*ZmYS3*, *ZmTOM2*, *ZmNRAMP1*) are significantly elevated in roots under Fe deficiency (Nozoye et al., 2013; Zanin et al., 2017). Moreover, Fe deficiency-inducible genes, such as *ZmTOM1*, *ZmDMAS1*, and *ZmIRT1*, are upregulated in the roots of *ys1* and *ys3* mutant under Fe-sufficient condition, suggesting that *ys1* and *ys3* plants are Fe-deficient during growth in the presence of Fe (Nozoye et al., 2013). Another research showed that *ZmYS3* which was identified by the linkage analysis in the Ye478 × Wu312 RIL population on the supply of Fe(III) (Xu et al., 2022), was only expressed in the roots and displayed a significant regulation under Fe deficiency.

In addition, ZmYS1 has a broad specificity for both metals and ligands, including Fe(III)-PS, Fe(III)-NA, Fe(II)-NA, Zn(II)-PS and Cu(II)-PS (Roberts et al., 2004; Schaaf et al., 2004; Murata et al., 2006; Harada et al., 2007). However, ZmYS1 protein may transport Zn and Cu at different rates, depending on the amounts of complexes (Roberts et al., 2004; Schaaf et al., 2004). *YSL-like* genes that are homologs of *ZmYS1* in rice, such as *AtYSL1* and *OsYSL2*, are reported to transport metals as NA complexes, including Zn(II)-NA, Cu(II)-NA, Fe(II)-NA and Mn(II)-NA (DiDonato et al., 2004; Koike et al., 2004; Jean et al., 2005; Ishimaru et al., 2010). In this study, we found that Fe deficiency substantially enhanced the uptake efficiency of Cu and Zn. This may be due to the increased amount of Cu(II)-PS and Zn(II)-PS complexes taken up by metal transporters that could be encoded by Fe deficiency-induced genes in roots, such as *ZmYS1*.

### Some Genes in Strategy I Plants May Be Involved in Fe Deficiency Tolerance in Maize

In this study, important genes involved in the regulation of Fe homeostasis in strategy I plants, including *ZmEIN2*, *ZmEIL1*, *ZmFIT*, *ZmPYE*, *ZmILR3*, and *ZmBTS*, were identified within the genomic regions detected by QTL analysis (Table 1). In *Arabidopsis*, the upstream regulator that controls about 50% of the Fe-regulated genes *via* direct promoter binding acts as a direct transcriptional activator of key genes involved in the Fe regulatory network, including *PYE* and *BTS* (Tissot et al., 2019; Gao et al., 2020; Lei et al., 2020). In *Arabidopsis thaliana*, a transcriptional regulatory network (PYE regulatory network) has a role in assuring the redistribution of already imported Fe (Long et al., 2010). ILR3 is involved in the regulation of *PYE* and the bHLH subgroup Ib genes (von der Mark et al., 2021). ILR3 represses the expression of several genes involved in Fe homeostasis *via* dimerization with PYE and direct binding to their promoters (Riaz and Guerinot, 2021). *Arabidopsis* BRUTUS-LIKE E3 ligases negatively regulate Fe uptake by targeting transcription factor FIT for recycling (Rodríguez-Celma et al., 2019). BRUTUS (BTS) could integrate local and long-distance iron signaling pathways, might negatively affect the PYE network (Cointry and Vert, 2019). Xu et al. (2022) found that *ZmPYE* was significantly induced by Fe deficiency in the shoots and roots in maize. In this study, *ZmBTS* displayed a greater upregulation in Fe-deficient shoots than in Fe-deficient roots of maize inbred lines, and a greater regulation in Fe-efficient inbred line rather than in Fe-inefficient inbred line (Figures 9C,D). Interestingly, *ZmPYE* reported in our other study and *ZmBTS* analyzed in this research showed the similar expression pattern between the Fe-efficient and Fe-inefficient genotypes. These two key genes (*PYE* and *BTS*) in the PYE regulatory system, whose functions have been verified in strategy I plants, may play an important role in the redistribution of already imported Fe to adapt to low Fe stress in maize. Our results also indicated that *ZmILR3* was Fe deficiency-inducible in shoots (Figure 9F). These findings implicate that the PYE regulatory network may participate in the mechanism underlying Fe deficiency stress tolerance in maize.

In addition, FIT is another regulatory hub for integrating signals from multiple signaling pathways, such as ethylene, nitric oxide, and reactive oxygen species (Brumbarova et al., 2015). Under Fe deficiency, ethylene is implicated in the activation of some Fe-related genes. EIN2 C-terminal fragment (EIN2-C) released upon cleavage indirectly triggers EIN3 and EIL transcription factors (Dolgikh et al., 2019). We also found that *ZmEIN2* was significantly induced by low Fe stress in maize (Figure 9E). EIN3/EIL binds with FIT, leading to positive regulation of the Fe deficiency response and activation of Fe uptake genes under Fe starvation (Riaz and Guerinot, 2021). In addition, ethylene production upon Fe deficiency positively affects FIT protein expression levels. Through a physical interaction between FIT and EIN3/EIL1, proteasomal degradation of FIT is reduced (Lingam et al., 2011). More comprehensive studies are needed to further confirm how these genes are involved in tolerance to Fe deficiency in maize.

## Data Availability Statement

The original contributions presented in the study are included in the article/Supplementary Material, further inquiries can be directed to the corresponding author/s.

## Author Contributions

JX and XQ performed the experiments. JX, XQ, and XF analyzed the data. JX, XQ, and HZ wrote the original manuscript. FC modified the manuscript. FY designed the study and modified the manuscript. All authors contributed to the article and approved the submitted version.

## Conflict of Interest

The authors declare that the research was conducted in the absence of any commercial or financial relationships that could be construed as a potential conflict of interest.

## Publisher’s Note

All claims expressed in this article are solely those of the authors and do not necessarily represent those of their affiliated organizations, or those of the publisher, the editors and the reviewers. Any product that may be evaluated in this article, or claim that may be made by its manufacturer, is not guaranteed or endorsed by the publisher.

## Footnotes

1
http://www.maizeGDB.org/

2
https://www.gramene.org/
