# Supplementary material for: Mapping of the Quantitative Trait Loci and Candidate Genes Associated With Iron Efficiency in Maize
Source: Front Plant Sci. 2022 Apr 22;13:855572. doi: 10.3389/fpls.2022.855572 (PMC9072831; doi:10.3389/fpls.2022.855572)
Supplement: Supplementary file 1 [file Data_Sheet_1.docx]

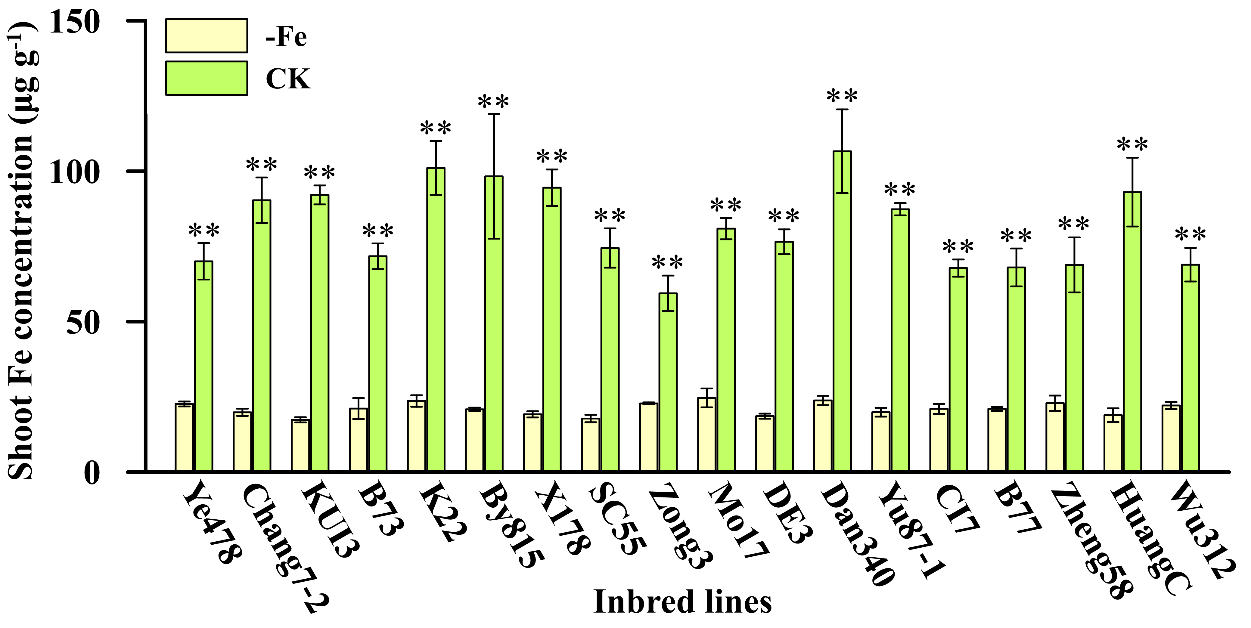


**Figure S1** Shoot Fe concentration of eighteen maize inbred lines under Fe-deficient [-Fe: 0.6 μmol L^-1^ Fe(II)-2,2’-bipyridyl] and Fe-sufficient [CK: 350 μmol L^-1^ Fe(II)-EDTA] conditions in Experiment 1. * and ** indicate significant differences between the -Fe and CK treatments at *p* ˂ 0.05 and *p* ˂ 0.01, respectively.


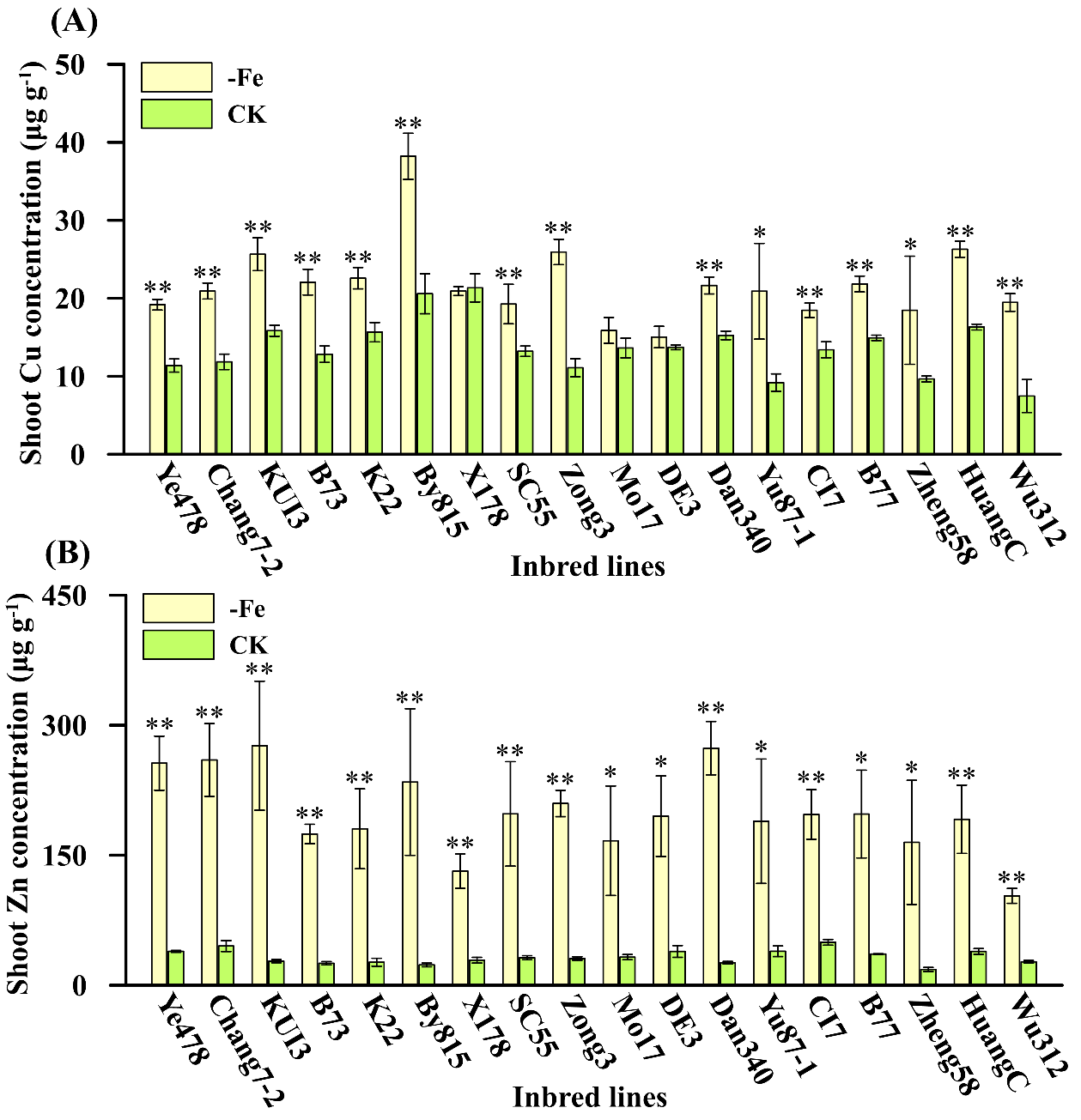


**Figure S2** Cu and Zn concentration (μg g^-1^) in the shoots of eighteen maize inbred lines under Fe-deficient [-Fe: 0.6 μmol L^-1^ Fe(II)-2,2’-bipyridyl] and Fe-sufficient [CK: 350 μmol L^-1^ EDTA-Fe(II)] conditions in Experiment 1. * and ** indicate significant differences between the -Fe and CK treatments at *p* ˂ 0.05 and *p* ˂ 0.01, respectively.


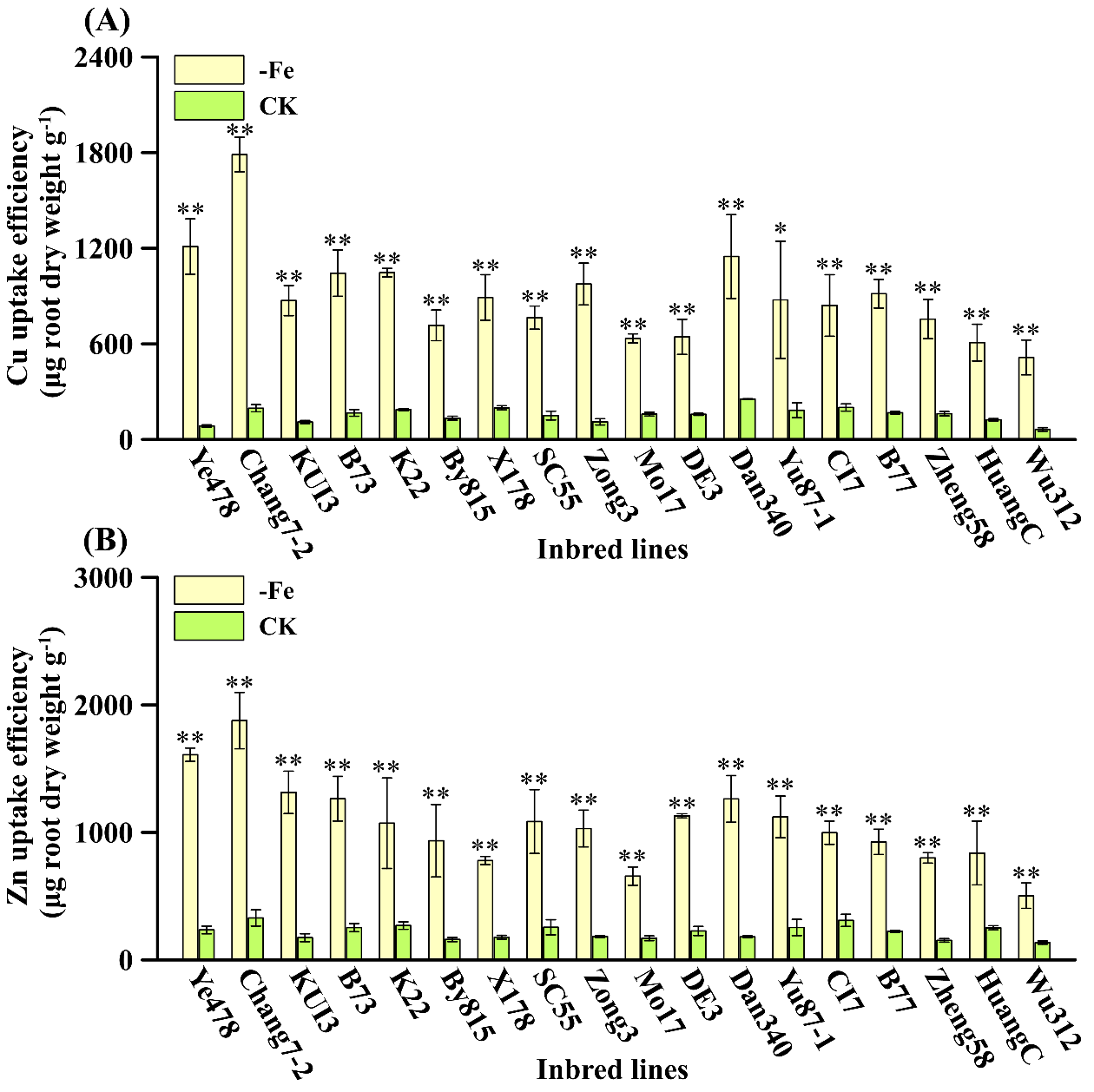


**Figure S3** Cu and Zn uptake efficiencies (μg root dry weight g^-1^) of eighteen maize inbred lines under Fe-deficient [-Fe: 0.6 μmol L^-1^ Fe(II)-2,2’-bipyridyl] and Fe-sufficient [CK: 350 μmol L^-1^ EDTA-Fe(II)] conditions in Experiment 1. * and ** indicate significant differences between the -Fe and CK treatments at *p* ˂ 0.05 and *p* ˂ 0.01, respectively.
